# The Oral Findings and Dental Management of Patients with West Syndrome: A Case Series and Literature Review

**DOI:** 10.3390/jcm14072494

**Published:** 2025-04-06

**Authors:** Jacobo Limeres-Posse, Carolina Muñoz-Navarro, Eliane García-Mato, Lucía Sande-López, Márcio Diniz-Freitas, Pedro Diz-Dios, Berta Rivas-Mundiña

**Affiliations:** Medical-Surgical Dentistry Research Group, Health Research Institute of Santiago de Compostela, School of Medicine and Dentistry, Santiago de Compostela University, 15705 Santiago de Compostela, Spain; carolinaalejandra.munoz@rai.usc.es (C.M.-N.); eliane.garma@gmail.com (E.G.-M.); luciasande.lopez@usc.es (L.S.-L.); pedro.diz@usc.es (P.D.-D.); berta.rivas@usc.es (B.R.-M.)

**Keywords:** West syndrome, epilepsy, oral manifestations, dentistry, dental care

## Abstract

**Objectives**: West syndrome (WS) is a rare disorder with an estimated prevalence of 1 in 4000 live births, characterized by infantile spasms, hypsarrhythmia, and psychomotor developmental impairment. The available information on dental care forWS patients remains limited. The aim of this study was to describe oral manifestations and dental management in a series of WS patients. **Methods:** Fourteen patients diagnosed with WS were evaluated, including 10 males and 4 females, aged 12–41 years. Medical and dental variables were collected for all patients. **Results:** The most frequent oral findings were poor oral hygiene (64.2%), gingivitis (64.2%), dental caries (57.1%), and bruxism/tooth wear (28.5%). Only one patient had dental fractures (due to trauma), and none exhibited drug-induced gingival enlargement. Initial dental treatment was carried out under general anesthesia in 42.3% of the patients. However, following desensitization, half of the patients showed improved behavior and were ultimately treated using non-pharmacological behavioral support techniques. **Conclusions:** This series represents the largest published to date on the dental aspects of WS. Dental treatment needs of WS patients are considerable, and their management is primarily determined by the degree of epilepsy control, the presence of comorbidities, and the level of cooperation. Nevertheless, these patients may benefit from desensitization strategies to improve their behavior. As subsequent sessions were conducted, the behavior of 1 in every 3 initially non-compliant patients showed significant improvement.

## 1. Introduction

In 1841, the English surgeon William James West described in a letter published in The Lancet a distinctive form of epilepsy that had first manifested in his own son at 8 months of age. This condition was characterized by repeated episodes of flexion and relaxation movements of the head, lasting 2 to 3 min, repeated throughout the day. He termed this phenomenon “infantile spasms” [1]. In 1960, the eponym “West Syndrome” (WS) was formally introduced [2] for a condition characterized by a diagnostic triad of infantile spasms, hypsarrhythmia, and developmental milestone loss [3].

The International League Against Epilepsy (ILAE) proposed a classification of epilepsy syndromes based on the age of onset, electroclinical features, management strategies, and prognostic implications, with its latest update in 2022 [4]. Within the category of epilepsy syndromes with onset in neonates and infants, the Infantile Epileptic Spasms Syndrome (IESS) encompasses both WS—when hypsarrhythmia is confirmed, regardless of developmental trajectory—and Infantile Spasms Syndrome—when spasms are confirmed, regardless of the electroencephalographic pattern [5].

The estimated incidence of IESS is 3 cases per 10,000 live births, although geographical variations have been reported, with higher rates observed in Nordic countries and a similar distribution across both sexes [6]. Although diagnosis is based on clinical and electroencephalographic findings, in 70% of cases the etiology is determined through neuroimaging, particularly MRI [7]. Spasms typically appear before 24 months of age, with a peak incidence between 3 and 12 months, coinciding with a slowing, cessation, or regression of development [4]. These spasms are brief tonic contractions, usually lasting less than 3 s, and predominantly affect axial muscles during wakefulness [4]. Hypsarrhythmia is an abnormal pattern of brain activity reflected in the electroencephalogram, characterized by chaotic waveforms, very slow and sharp waves, and epileptiform discharges [8]. Neuropsychomotor developmental delay is a significant lag and deviation from typical developmental trajectories, where a child demonstrates delayed achievement of expected skills compared to same-age peers across multiple domains such as motor function, cognitive abilities, language development, and social–emotional skills [9]. The severity of developmental delay has been correlated with the frequency and intensity of spasms [9], though some authors have suggested that neurodevelopmental sequelae may be independent of seizure progression [4].

Traditionally, IESS has been classified as symptomatic and cryptogenic [7], with the later addition of the idiopathic category [10]. In patients with an unknown etiology, a neurotypical development prior to the onset of symmetrical spasms enables thedifferentiation of the idiopathic form from the cryptogenic form [5]. However, in 2017, the ILAE proposed a more comprehensive classification based on etiology, subdividing epilepsies into structural, genetic, metabolic, infectious, immunological, and unknown [11]. Although the pathophysiology of IESS remains largely unclear, it has been associated with a nonspecific insult occurring during a critical period of the ontogenetic development of the brain [12], as well as dysfunction of the hypothalamic–pituitary–adrenal axis leading to the overproduction of corticotropin-releasing hormones [13].

In general, IESS is considered a syndrome with a poor prognosis [7], as it is associated with a high mortality rate and frequently evolves into other forms of epilepsy, such as Lennox–Gastaut syndrome (~30%) or drug-resistant focal epilepsies [4]. While epileptic spasms may persist in some patients, particularly those with genetic or structural encephalopathies, they may resolve effectively with treatment in others [4]. Among the sequelae of IESS are moderate-to-severe neurodevelopmental support needs, which affects approximately 60% of patients [14]. Cryptogenic etiology and early initiation of treatment (within the first 4 months) are considered factors that improve prognosis [14].

First-line treatment for IESS includes adrenocorticotropic hormone (ACTH) [15]. Corticosteroids (Go) and vigabatrin—a GABA-transaminase inhibitor [16]—as well as various combinations of these three drugs [17]. In some cases, topiramate, levetiracetam, or the ketogenic diet are also employed to manage the spasms [18].

Although WS has been extensively studied from a medical standpoint, there have been few studies published on its dental aspects [19,20,21,22,23,24,25,26,27,28,29], most of which consist of isolated clinical case reports. The research hypothesis is that patients with WS may present oral manifestations particularly prevalent in relation to epilepsy and its pharmacological treatment [30]. Their dental management can be complex, especially in patients with refractory epilepsy [31]. Moreover, patients with WS often exhibit an atypical neurodevelopmental pattern, such as motor difficulties, communication barriers, and behavioral issues, which can pose a challenge in the dental setting [32]. The aim of this study is to describe the oral manifestations and the unique considerations in dental treatment in a cohort of patients with WS.

## 2. Materials and Methods

A retrospective review of the medical records of patients who attended the Special Care Dentistry Unit at the Santiago de Compostela University (Spain) over the past 15 years was conducted. Patients with a confirmed diagnosis of WS were selected based on this single inclusion criterion. Informed consent was obtained from all subjects involved in the study or their legal representatives. Ethical approval was not necessary according to the regulations of the authors’ university.

The study group comprised 14 patients, 4 females and 10 males, with a mean age of 22.8 years (range, 12–41 years). Medical data (epilepsy control, comorbidities, and pharmacological treatment) and oral health data (oral manifestations and behavior in the dental setting) were extracted for all individuals.

Patients with epilepsy were defined as those with a confirmed diagnosis of epilepsy or those receiving anticonvulsant medication. Epilepsy was considered “controlled” when the patient had been seizure-free for the preceding 6 months, and “uncontrolled” if at least one seizure had occurred within the past 6 months. The “unspecified” category was used when this information was not documented in the medical record.

Comorbidities were recorded with the name of each condition or its corresponding eponym, including neuro- and/or psychomotor delay, Lennox–Gastaut syndrome, or autism spectrum disorder, among others. The pharmacological treatment current at the time of the review of the medical records was also noted; pharmacological agents were classified as anticonvulsants and other frequently used medications in WS patients (e.g., ACTH or corticosteroids).

Oral examination was performed in the dental office, unaided (with a mirror and probe), using physical restraint when necessary. Oral health data were categorized into three groups according to the type of tissue involved—teeth, soft tissue, and bone. For the teeth, the following were recorded: poor oral hygiene (visible dental plaque without the use of disclosing agents), white spot lesions (ICDAS = 2), active caries (ICDAS > 2), bruxism (observed during the examination or reported by the caregiver), dental attrition, delayed dental eruption (more than 1 year delayed compared to chronological age), dental malposition, and enamel hypoplasia (regardless of the type and number of teeth involved). The soft tissue-related variables included gingivitis, gingival enlargement, and lingual malposition. The bone-related variables included high-arched palate and anterior open bite. Oral health data were recorded for all patients by 2 examiners from the same unit, who undergo a calibration process periodically (once a year). In case of discrepancies, a third senior staff member (JLL or PDD) was consulted.

Regarding the behavior of WS patients in the dental clinic and the behavioral support techniques applied, the following variables were recorded: cooperative behavior (the patient was compliant and no techniques were needed to control them) and cooperation difficulties (when non-pharmacological and pharmacological behavioral support methods were required). Non-pharmacological methods included behavior shaping through successive approximation, desensitization, and protective stabilization. Pharmacological behavioral support was used exclusively for dental treatment sessions and involved premedication (diazepam or midazolam orally), sedation (midazolam intranasally), and general anesthesia. The need for, and if applicable, the selection of behavioral support techniques was determined by a senior staff member (JLL or PDD).

## 3. Results

In the present cohort, 11 patients (78.5%) had epilepsy and/or received anticonvulsant medication. Epilepsy was poorly controlled in nineof these patients (64.2%). Regarding comorbidities, the most prevalent was neuro- and/or psychomotor delay (92.8%), followed by progression to Lennox–Gastaut syndrome (42.8%) and tetraparesis/limb atrophy (28.5%). The most commonly prescribed medications were anticonvulsants, particularly valproic acid (78.5%), topiramate (28.5%), and clobazam (28.5%). These results are detailed in Table 1.

**Figure 1:**
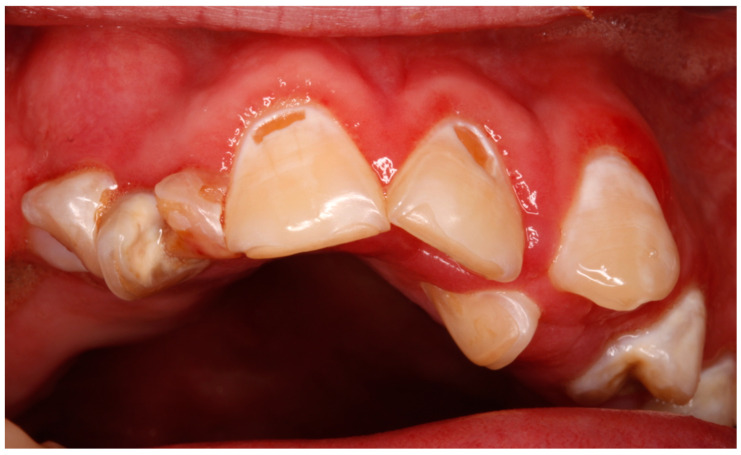
Neglected oral health status in a patient with West syndrome, with poor oral hygiene, dental caries, and gingivitis.

The most common dental findings included poor oral hygiene (64.2%), dental caries (57.1%) (Figure 1), and bruxism/attrition (28.5%). In terms of oral health status, 64.2% of patients had gingivitis. Only one maxillary anomaly was recorded (anterior open bite). These findings are summarized in Table 2.

Cooperative behavior was recorded in fivepatients (35.7%) during the initial dental treatment session which is always restricted to non-invasive or minimally invasive procedures—for which local anesthesia injection is not required. Among the remaining ninepatients, physical restraint was required for 1, conscious sedation for 2, and general anesthesia for the remaining six (42.8% of the entire cohort). As subsequent sessions were conducted, the behavior of threeinitially non-compliant patients showed significant improvement.

## 4. Discussion

This study presents a comprehensive description of oral findings and the unique aspects of dental treatment in a cohort of 14 adolescent and young adult patients with WS. To the best of our knowledge, this is the largest series published to date (as can be seen in the Supplementary Material, it includes 41% of the total published cases on oral findings in patients with WS). In the dental literature, only one series, published in 2014, included 8 Brazilian children with WS [22], along with several isolated clinical cases, mostly pediatric, collectively comprising a total of 20 patients [19,20,21,22,23,24,25,26,27,28,29]. In the current series, there was a male predominance with a ratio of 2.5:1, compared to 1.5:1 in previously published dental reports; although epidemiologically no sex bias has been definitively confirmed [6], some authors have suggested a slight male predominance [33].

In the majority of patients in the present cohort, WS had progressed to drug-resistant focal epilepsy or Lennox–Gastaut syndrome, consistent with previous studies [4]. While the case definition of WS no longer requires evidence of developmental delay prior to the onset of spasms [5], all but one patient in this series exhibited some degree of neuro- and/or psychomotor developmental delay, which may influence communication with the dentist and impact the level of cooperation in the dental office. Tetraparesis or limb atrophy was documented in fourpatients in this series, a finding that affects approximately 20% of WS patients [34], potentially compromising autonomy in maintaining proper oral hygiene and complicating transfer to the dental chair. The characteristics of epilepsy and comorbidities in WS patients reported in the dental literature are detailed in Tables S1 and S2.

The most commonly prescribed anticonvulsants in the present cohort may induce side effects of particular relevance in the dental setting, such as hematotoxicity (e.g., neutropenia or thrombocytopenia) associated with valproic acid [35], and behavioral disturbances (e.g., impulsivity or aggressiveness) induced by valproic acid or topiramate [36]. In WS patients reported in the dental literature, the most commonly used medications were (Table S3) nitrazepam, which has been linked to drowsiness and sialorrhea [37]; vigabatrin, which may cause irritability [9]; and ACTH, whose adverse effects include immunosuppression [38].

To date, no specific oral manifestations of WS have been definitively identified. The most prevalent findings in the present cohort were poor oral hygiene, dental caries, gingivitis, and bruxism. It has been noted that maintaining adequate oral hygiene standards in WS patients is challenging due to cognitive impairment, communication difficulties, and limited manual dexterity [39]. Furthermore, gingival enlargement induced by some anticonvulsants can promote plaque accumulation and bleeding, thereby complicating the performance of proper oral hygiene [30].

In the dental literature, 70% of the reported cases have been documented to have dental caries [19,21,22,23,24,25,26,27,28,29] (Table S4). Poor oral hygiene increases the risk of caries, which can be further exacerbated by carbohydrate-rich diets [19] and the sugar content of certain medications [23].

In the present series, gingivitis was diagnosed in over 60% of the patients; however, no cases of gingival enlargement were recorded. In contrast, the prevalence of gingivitis in the dental literature is reported to be 25% [21,24,26,29], while the prevalence of drug-induced gingival enlargement associated with antiepileptic drugs is another 25% [19,24,27,35]. In addition to phenytoin, gingival enlargement has also been linked to other antiepileptic drugs used by the patients in this series, including phenobarbital, clobazam, valproic acid, levetiracetam, and topiramate [40]. In some patients with WS, generalized gingival inflammation due to plaque accumulation has been described [21], which in certain cases has been reported as generalized gingival enlargement [25]. Consequently, it is possible that some of the cases diagnosed as gingivitis in this series, based on the presence of dental plaque and the clinical appearance of the lesion, may actually represent gingival enlargements associated with antiepileptic drug use.

Bruxism was diagnosed in approximately 30% of patients with WS, both in this series and in cases reported in the dental literature [21,23,24,27], compared to 21% in the general European population [41]. Bruxism has been closely associated with certain types of childhood epileptic encephalopathy [42] and is more prevalent in children with refractory epilepsy than in well-controlled cases [43].

Dental fractures were detected in only one patient. This finding has not been previously reported in the dental literature on WS. Generally, the prevalence of dental fractures in patients with epilepsy is low (~2%) and accounts for only 11% of injuries caused by seizures [44]. These dental traumas are almost exclusively associated with generalized tonic–clonic seizures [45], making their expected frequency in patients with IESS likely even lower.

In the present series, no cases of high-arched palate were found, in contrast to the 35% prevalence reported in the dental literature for patients with WS [21,22,23,24,25,26,27,28]. A high-arched palate is a characteristic commonly observed in certain neurodegenerative disorders associated with epilepsy, such as Rett syndrome [46]. It has been suggested that this condition may arise from disruptions in palatogenesis, although its precise etiology remains unclear [47]. The true prevalence of high-arched palate is yet to be established, as objective measurements of the palatal vault have been available for nearly 60 years [48], but more precise and reproducible measurement techniques are required to accurately define “normal palatal morphology”.

Regarding patient cooperation, good behavior was recorded in approximately one-third of the patients, compared to one-fourth in the dental literature on WS [21,26,27]. General anesthesia was necessary in over 40% of patients, both in the present series and in previously reported cases [20,21,23,25,29]. Clinical guidelines for providing dental treatment in patients with poorly controlled epilepsy include pharmacological sedation with oral or intravenous benzodiazepines, as well as the use of general anesthesia [31]. In such cases, in addition to the frequency and severity of epileptic seizures, the need for general anesthesia may be determined by the extent of required dental treatment, acute dental processes, or in instances of severe intellectual disability that significantly impair cooperation [31]. Desensitization techniques have proven effective in dental settings for patients with intellectual disabilities and communication difficulties [49], and their efficacy has also been confirmed in the present series for patients with WS who initially received treatment under general anesthesia.

This study has several limitations that hinder direct comparison with previously published cases. These limitations include the highly variable progression of WS (ranging from refractory epilepsy to complete resolution of epilepsy), the lack of universally applied definitions to describe oral findings, and the absence of examiner calibration. Additionally, some variables, such as cooperation level, were not consistently recorded, and the behavioral support techniques employed may have been influenced by factors unrelated to the patients, such as access to general anesthesia.

## 5. Conclusions

The present series underscores the substantial dental treatment needs of patients with WS, emphasizing the necessity of implementing early oral hygiene and preventive measures. Some of these patients may benefit from desensitization strategies to facilitate behavioral support. Ultimately, the clinical progression of WS is highly variable, as evidenced by the fact that 2 patients in this series were able to complete orthodontic treatment.

## Figures and Tables

**Table 1:** TheMedical Characteristics of Patients with West Syndrome in the Present Series (*n* =14).

Medical Characteristics		Cases (%)
	Controlled	2 (14.2)
	Uncontrolled	9 (64.2)
	Resolved *	3 (21.4)
Epilepsy	Neuropsychomotor delay	13 (92.8)
	Lennox–Gastaut syndrome	6 (42.8)
	Tetraparesis	3 (21.4)
	Autism spectrum disorder	2 (14.2)
Comorbidities	Limb atrophy	1 (7.1)
	Scoliosis	1 (7.1)
Anticonvulsants		
	Valproic acid	11 (78.5)
	Topiramate	4 (28.5)
	Clobazam	4 (28.5)
	Clonazepam	3 (21.4)
	Phenobarbital	3 (21.4)
	Levetiracetam	3 (21.4)
	Lamotrigine	1 (7.1)
	Vigabatrin	0
Other medications		
	Baclofen	1 (7.1)
	Risperidone	1 (7.1)
	ACTH	0
	Corticosteroids	0

* No episodes for years and no anticonvulsant medication.

**Table 2:** Oral Findings in Patients with West Syndrome in the Present Series (*n* =14).

Oral Findings		Cases (%)
Dental anomalies		
	Poor oral hygiene	9 (64.2)
	Cavities	8 (57.1)
	Bruxism/attrition	4 (28.5)
	Dental malposition	2 (14.2)
	Enamel hypoplasia	2 (14.2)
	White spot lesion	1 (7.1)
	Dental fracture	1 (7.1)
	Abnormal tooth eruption	0
	Delayed tooth eruption	0
Soft tissues anomalies		
	Gingivitis	9 (64.2)
	Lingual malposition	1 (7.1)
	Gingival enlargement	0
Maxillary bones anomalies		
	Anterior open bite	1 (7.1)
	High palate	0

## Data Availability

The data that support the findings of this study are available on requestfrom the corresponding authors.

## Supplementary Materials

The following supporting information can be downloaded at: https://www.mdpi.com/article/10.3390/jcm14072494/s1, Table S1: Degree of Epilepsy Control in Patients with West Syndrome (Literature Review and Present Series); Table S2. Comorbidities in Patients with West Syndrome (Literature Review and Present Series); Table S3. Prescription of Anticonvulsants and Other Medications in Patients with West Syndrome (Literature Review and Present Series); Table S4. Oral Findings in Patients with West Syndrome (Literature Review and Present Series); Table S5. The Behavior of Patients with West Syndrome in the Dental Office and Applied Behavior Support Techniques (Literature Review and Present Series).
