# Supplementary material for: The Oral Findings and Dental Management of Patients with West Syndrome: A Case Series and Literature Review
Source: J Clin Med. 2025 Apr 6;14(7):2494. doi: 10.3390/jcm14072494 (PMC11989459; doi:10.3390/jcm14072494)
Supplement: Supplementary file 1 [file jcm-14-02494-s001.zip › jcm-3564637-supplementary.pdf]

Table S1: Degree of Epilepsy Control in Patients with West Syndrome (Literature Review and Present Series)

**Author, year (number of cases)**	**EPILEPSY CHARACTERISTICS***			
	**Controlled Epilepsy**	**Uncontrolled Epilepsy**	**Not specified**	**Resolved****
Regis et al., 2009 (N=1)			1	
Bussell, 2010 (N=1)		1		
Khatri et al., 2014 (N=2)			2	
Dantas-Neta et al., 2014 (N=8)			8	
Badnaware et al.,2017 (N=1)	1			
Nacamuraetal., 2018 (N=1)			1	
Della Vella et al., 2019 (N=1)			1	
Akbar et al., 2020 (N=1)			1	
Ferreira do Amaral et al., 2020 (N=2)		1	1	
Goswami et al., 2021 (N=1)			1	
Mehrotra et al., 2024 (N= 1)		1		
Present series (N=14)	2	9		3
TOTAL (N= 34)	3 (8.8%)	12 (35.2%)	16 (47.0%)	3 (8.8%)

*Diagnosis of epilepsy or users of anticonvulsant drugs; **No episodes for years and no anticonvulsant medication (resolved epilepsy)

Table S2. Comorbidities in Patients with West Syndrome (Literature Review and Present Series)

**Author, year (number of cases)**	**COMORBIDITIES**						
	**Neuro-psycho-motordelay**	**Lennox-GastautSyndrome**	**Tetraparesis**	**Limbatrophy**	**Autismspectrumdisorder**	**Scoliosis**	**Neonatal jaundice**
Regis et al., 2009 (N= 1)	1						
Bussell, 2010 (N= 1)							
Khatri et al., 2014 (N= 2)	2						1
Dantas-Neta et al., 2014 (N= 8)					1		1
Badnaware et al., 2017 (N= 1)				1			
Nacamuraetal., 2018 (N= 1)	1		1			1	
Della Vella et al., 2019 (N= 1)							
Akbar et al., 2020 (N= 1)	1			1			
Ferreira do Amaral et al., 2020 (N= 2)	2				1		
Goswami et al., 2021 (N= 1)	1						
Mehrotra et al., 2024 (N= 1)	1			1			
Present series (N=14)	13	6	3	1	2	1	
TOTAL (N= 34)	22 (67.6%)	6 (17.6%)	4 (11.7%)	4 (11.7%)	4 (11.7%)	2 (5.8%)	2 (5.8%)

Table S3. Prescription of Anticonvulsants and Other Medications in Patients with West Syndrome (Literature Review and Present Series)

**Author, year (number of cases)**	Regis et al., 2009 (N= 1)	Bussell, 2010 (N= 1)	Khatri et al., 2014 (N= 2)	Dantas-Neta et al., 2014 (N= 8)	Badnaware et al., 2017 (N= 1)	Nacamuraetal., 2018 (N= 1)	Della Vella et al., 2019 (N= 1)	Akbar et al., 2020 (N= 1)	Ferreira do Amaral et al., 2020 (N= 2)	Goswami et al., 2021 (N= 1)	Mehrotra et al., 2024 (N= 1)	Present series N=14	TOTAL N= 34 (%)
**ANTIEPILEPTICS**													
Valproicacid	1	1	2	4		1						11	20 (58.8%)
Clonazepam		1	1	1					1			3	7 (20.5%)
Phenobarbital	1			2								3	6 (17.6%)
Topiramate		1	1									4	6 (17.6%)
Levetiracetam					1						1	3	5 (15.6%)
Clobazam									1			4	5 (14.7%)
Vigabatrin		1		2			1						4 (11.7%)
Lamotrigine		1							1			1	3 (8.8%)
Carbamazepine								1					1 (2.9%)
**OTHER DRUGS**													
Nitrazepam				3		1	1						5 (14.7%)
ACTH							1		2	1			4 (11.7%)
Baclofen	1	1										1	3 (8.8%)
Risperidone				1								1	2 (5.8%)

Table S4. Oral Findings in Patients with West Syndrome (Literature Review and Present Series)

**Author, year (number of cases)**	Regis et al., 2009 (N= 1)	Bussell, 2010 (N= 1)	Khatri et al., 2014 (N= 2)	Dantas-Neta et al., 2014 (N= 8)	Badnaware et al., 2017 (N= 1)	Nacamuraetal., 2018 (N= 1)	Della Vella et al., 2019 (N= 1)	Akbar et al., 2020 (N= 1)	Ferreira do Amaral et al., 2020 (N= 2)	Goswami et al., 2021 (N= 1)	Mehrotra et al., 2024 (N= 1)	Present series N=14	TOTAL N= 34 (%)
**DENTAL ANOMALIES**													
Cavities	1		2	5	1	1	1		1	1	1	8	22 (64.7%)
Poor oral hygiene						1		1			1	9	12 (35.2%)
Bruxism/Attrition		1	2		1	1			2			4	11 (32.3%)
Abnormal tooth eruption						1	1		2				4 (11.7%)
Dental malposition	1										1	2	4 (11.7%)
Enamel hypoplasia		1			1							2	4 (11.7%)
White spot lesion	1						1					1	3 (8.8%)
Delayed tooth eruption	1	1				1							3 (8.8%)
Dental fracture												1	1 (2.9%)
**SOFT TISSUES ANOMALIES**													
Gingivitis			2			1		1			1	9	14 (41.1%)
Gingival enlargement	1					1	1		2				5 (14.7%)
Lingual malposition				3		1						1	5 (14.7%)
**MAXILLARY BONES ANOMALIES**													
High palate			2	2	1	1	1	1	2	1			11 (32.3%)
Anterior open bite					1	1						1	3 (8.8%)

Table S5. Behavior of Patients with West Syndrome in the Dental Office and Applied Behavior Support Techniques (Literature Review and Present Series)

**Author, year (number of cases)**	Regis et al., 2009 (N= 1)	Bussell, 2010 (N= 1)	Khatri et al., 2014 (N= 2)	Dantas-Neta et al., 2014 (N= 8)	Badnaware et al., 2017 (N= 1)	Nacamura et al., 2018 (N= 1)	Della Vella et al., 2019 (N= 1)	Akbar et al., 2020 (N= 1)	Ferreira do Amaral et al., 2020 (N= 2)	Goswami et al., 2021 (N= 1)	Mehrotra et al., 2024 (N= 1)	Present series N=14	TOTAL N= 26* (%)
**COOPERATIVE BEHAVIOR**													
			1	NS				1	1			5 (3**)	8 (30.7%)
**COOPERATION DIFFICULTIES*** **													
	1	1	1	NS	1	1	1		1	1	1	9	18 (69.2%)
**BEHAVIOR SUPPORT TECHNIQUES**													
Protective stabilization	1					NS			1	NS		1	3 (11.5%)
Conscious sedation						NS	1			NS		2	3 (11.5%)
General anesthesia		1	1		1	NS	1			NS	1	6***	11 (42.3%)

*N: Specified in only 26 out of 34 cases; NS: Not specified; **Three patients exhibited non-cooperative behavior at the beginning of treatment, which improved as sessions progressed; *** In some patients, the indication for general anesthesia is more justified by poor epilepsy control than by the patient's level of cooperation.
